# Eye-gaze control of a wheelchair mounted 6DOF assistive robot for activities of daily living

**DOI:** 10.1186/s12984-021-00969-2

**Published:** 2021-12-18

**Authors:** Md Samiul Haque Sunny, Md Ishrak Islam Zarif, Ivan Rulik, Javier Sanjuan, Mohammad Habibur Rahman, Sheikh Iqbal Ahamed, Inga Wang, Katie Schultz, Brahim Brahmi

**Affiliations:** 1 Department of Computer Science, University of Wisconsin-Milwaukee, Milwaukee, WI 53211 USA; 2 Department of Computer Science, Marquette University, Milwaukee, WI 53233 USA; 3 Mechanical Engineering Department, University of Wisconsin-Milwaukee, Milwaukee, WI 53211 USA; 4 Department of Rehabilitation Sciences and Technology, University of Wisconsin-Milwaukee, Milwaukee, WI 53211 USA; 5 Clement J. Zablocki VA Medical Center, Milwaukee, WI 53295 USA; 6 Department of Electrical and Computer Engineering, Miami University, Oxford, OH 45056 USA

**Keywords:** Assistive robot, 6DoF, Eye-gaze control, Wheelchair, Motor dysfunction, Wheelchair mounted robot, Activities of daily living

## Abstract

**Background:**

Building control architecture that balances the assistive manipulation systems with the benefits of direct human control is a crucial challenge of human–robot collaboration. It promises to help people with disabilities more efficiently control wheelchair and wheelchair-mounted robot arms to accomplish activities of daily living.

**Methods:**

In this study, our research objective is to design an eye-tracking assistive robot control system capable of providing targeted engagement and motivating individuals with a disability to use the developed method for self-assistance activities of daily living. The graphical user interface is designed and integrated with the developed control architecture to achieve the goal.

**Results:**

We evaluated the system by conducting a user study. Ten healthy participants performed five trials of three manipulation tasks using the graphical user interface and the developed control framework. The 100% success rate on task performance demonstrates the effectiveness of our system for individuals with motor impairments to control wheelchair and wheelchair-mounted assistive robotic manipulators.

**Conclusions:**

We demonstrated the usability of using this eye-gaze system to control a robotic arm mounted on a wheelchair in activities of daily living for people with disabilities. We found high levels of acceptance with higher ratings in the evaluation of the system with healthy participants.

## Backgrounds

In recent times, eye gaze has been introduced to control graphical user interfaces directly. Eye gaze-controlled interfaces have been used for people with a severe motor impairment who cannot use the alternative computer peripherals. In this research, we developed a control architecture for an eye-gaze control of a wheelchair-mounted assistive robot for activities of daily living (ADL) of individuals with upper limb impairment. Functional impairments of the Upper or Lower Extremities (ULE) are common in the elderly [1]. They are often secondary to conditions such as strokes, spinal cord injury (SCI), amyotrophic lateral sclerosis (ALS), Cerebral Vascular Accident (CVA), trauma, and occupational injuries [2]. Being a powerful tool for object manipulation and communication, the human Upper Extremity (UE) is the most evolved body part. Its functionality largely reflects our workability, and the quality of life heavily relies on the proper functioning of UE motion [3]. It is challenging for a person to do their daily activities due to physical mobility loss [4]. In recent decades the number of Individuals with UE disabilities has increased at an alarming rate.

Approximately 5,357,970 people, or 1.7% of the US population, currently live with central nervous system disorders, causing difficulty or inability to move the upper and lower portions of the body [5]. Around 353,000 Americans live with a spinal cord injury, and more than 795,000 US people suffer a cerebral vascular accident each year, resulting in significant deficits in upper and lower limb disabilities [5]. Among them, it is estimated that about two-thirds of cerebral vascular accident survivors suffer acute arm impairment [5, 6]. Loss of upper limb function significantly limits the independence of the affected person and everyday tasks' performance—a significant challenge for these people to do their daily activities without any external help.

Although a caregiver and assistive devices provide support for specific tasks, very few individuals use disabled adapted facilities [7, 8]; however, such facilities have restricted their mobility. In most ADLs, individuals with upper/lower extremities dysfunctions (IwULEDs) can not take care of themselves without significant help from others [9]. Thus, research on ADL and mobility assistance targeting this specific population is an immense need to improve the independence of those individuals and, therefore, reduce the burden of care for the family.

Modern technologies help people with disabilities improve their quality of life and do their daily activities using assistive robots/devices [10–13]. People with motor dysfunctionalities face several problems in carrying out ADL, including feeding, toileting, dressing, and bathing [14–18]. Usages of assistive technologies like a powered wheelchair and robotic hand to assist these people are on the rise [4]. Working with assistive technologies and upgrading them for better usage is becoming a newer trend [4] and caught the interest of clinics, research, and industry [19]. Different types of research work are going on to improve assistive technology [20–22]. In particular, assistive robotics appeared as an exciting research topic that can enhance people's quality of life with motor dysfunctionalities. The earlier powered wheelchair was controlled with a joystick [23–25], but people with musculoskeletal disease/injury could not use the hand-controlled joystick [26]. That is the reason why eye-gaze control has been explored [19, 27–32]. And researchers are improving the eye-gaze control of an assistive robot to help these impaired people's daily activities. This eye-gaze control of assistive robot technology aims to help mute, paralyzed, elderly, or confined patients so that they can communicate with assistive robots easily, do their daily living activities, and live independently [29].

Eye-tracking is a powerful means for assistive technologies for people with movement disabilities [28, 30, 33, 34]. An eye-tracking device combined with an assistive robot can increase users' speed of interaction and comfort [30, 35, 36]. Therefore, researchers are interested in developing an eye-gaze system interface for paralyzed or physically disabled people [27].

This study developed an eye-gaze control architecture and designed a user-friendly graphical user interface with virtual buttons to control wheelchair and wheelchair-mounted 6DoF robotic arms in cartesian mode. In our control, we added a feature to load the predefined path of the robot's end-effector to perform an activity of daily living repetitively, such as a feeding task. We also solved the inverse kinematics of a 6DoF robotic arm, named xArm [37] to achieve the research goal. This work will allow people with UE dysfunctions to manipulate both a wheelchair and a robotic assistive arm to ease their lives.

### Related works

Some recent works in assistive technologies and how assistive technologies are used to improve the mobility of individuals with a disability are described [4]. This work consists of diverse technology, including powered wheelchairs, prosthetic limbs, functional electrical stimulation, and wearable exoskeletons. This work tried to improve the existing assistive technology mechanics, improve the physical interface, and focus on sharing the control between the technology and the user who will use the system. Oculomotor behaviors, gaze control, smooth pursuit, saccades, and their interactions with vision are described in [38], emphasizing on past 25 years' literature review on eye movements. From this work, we got the general idea of how prediction, learning, and attention work with sensory signals and how these things contribute to effective eye movement operations. If the head movement was permitted during eye-tracking, it isn't easy to track the eye movement. A simple eye gaze tracker-based interface controls the wheelchair with free head movement described in [27]. A 3D orientation sensor is used in this eye-gaze estimation system to measure the head position and orientation. In this way, a user can keep a comfortable head pose or change head position while navigating the wheelchair. But the experimental result shows that this system is a little bit slower than other keyboard-based navigation systems.

In [28], an eye-tracking-based telerobotic arm system is proposed for tele-writing and drawing. Eight subjects were selected and trained for the experiment. This system's primary purpose is to use eye-tracking to operate a robotic arm and move the robotic arm with their gaze on a flat vertical screen for writing the word with a pen attached with the arm at the endpoint. Authors in [29] describe using a low-cost gaze tracking hardware (Gazepoint GP3 Eye Tracker) to visually draw some shapes, used as input for robot command. A custom algorithm and MATLAB are used to detect and process the command. A small humanoid robot (NAO) is used for this experiment. The robot works as a personal assistant for impaired people, and people can use an eye gaze tracking system to command the robot. An eye-tracking robotic system for controlling a wheelchair and doing some daily living activities (ADL) using a wheelchair-mounted hand-exoskeleton is explored in [30]. The system is named EMOHEX, which can help people with disabilities move the wheelchair and also hold an object using the robotic arm. A graphical user interface is developed in [31] to control the powered wheelchair. An eye tracker interacts with the system and helps people with upper limb disabilities do their daily living activities.

The work in [32] describes an intelligent user interface designed with an eye-tracking system named Display/Eye Tracker device Set (DETS). The system is based on the VHF + algorithm. Using this system, a wheelchair user can select a destination on a local map space, and after that, the wheelchair automatically started to go to that destination point. The objective of this work [19] is to use low-cost equipment and design a system where an assistive robotic arm can be used with the help of eye-tracking. The research aimed so that individuals can afford the system and use the system to control the robotic arm for reaching and grasping an object successfully.

## Methods

### Description of materials

#### Robotic arm

**Fig. 1:**
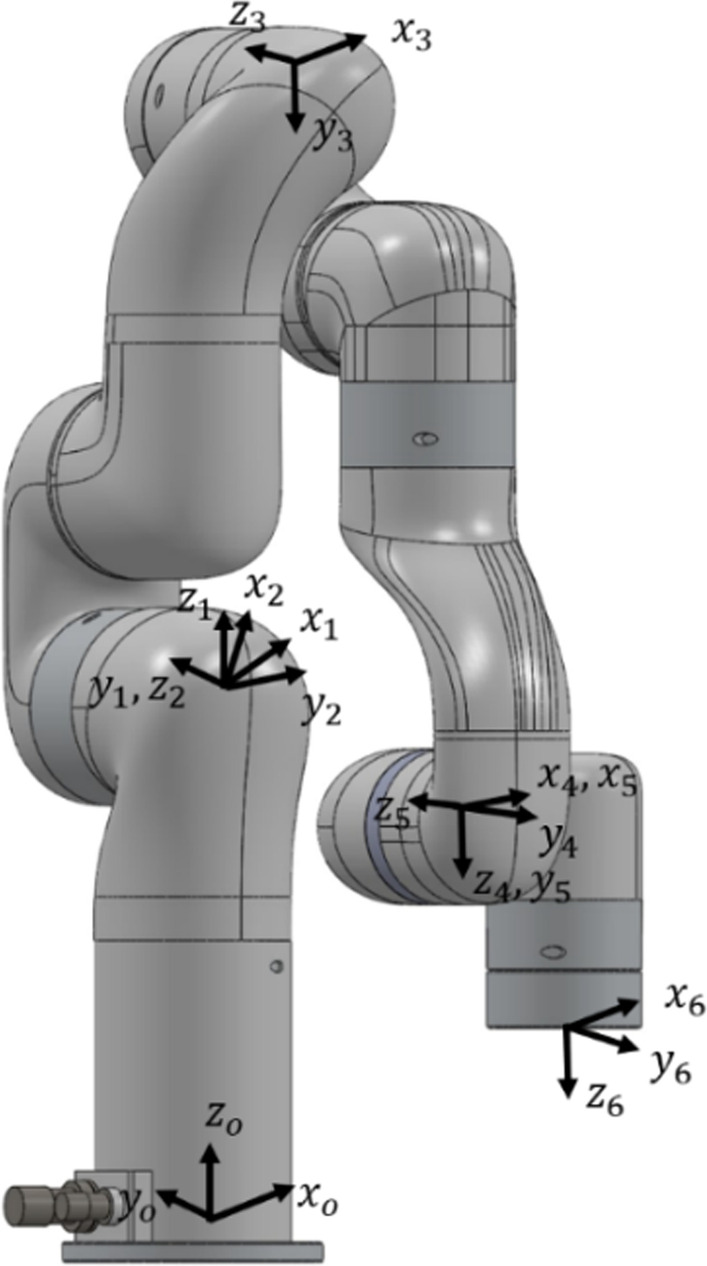
Joint coordinate system

In this research, xArm 6 from UFactory is used [37]. The xArm 6 is a versatile, 6 degree of freedom (DoF) robotic arm utilizing high-performance harmonic reducers and brushless motors to achieve a payload capacity of 5 kg and repeat position accuracy of ± 0.1 mm. It has RS-485 [39] communication mode, Modbus RTU protocol [40]. It has the position, speed, and force control for programmable gripping parameters with motor current, gripper position, video, and grip detection as feedback. Figure 1 shows the joint coordinate definition of xArm 6.

#### Powered wheelchair

**Fig. 2:**
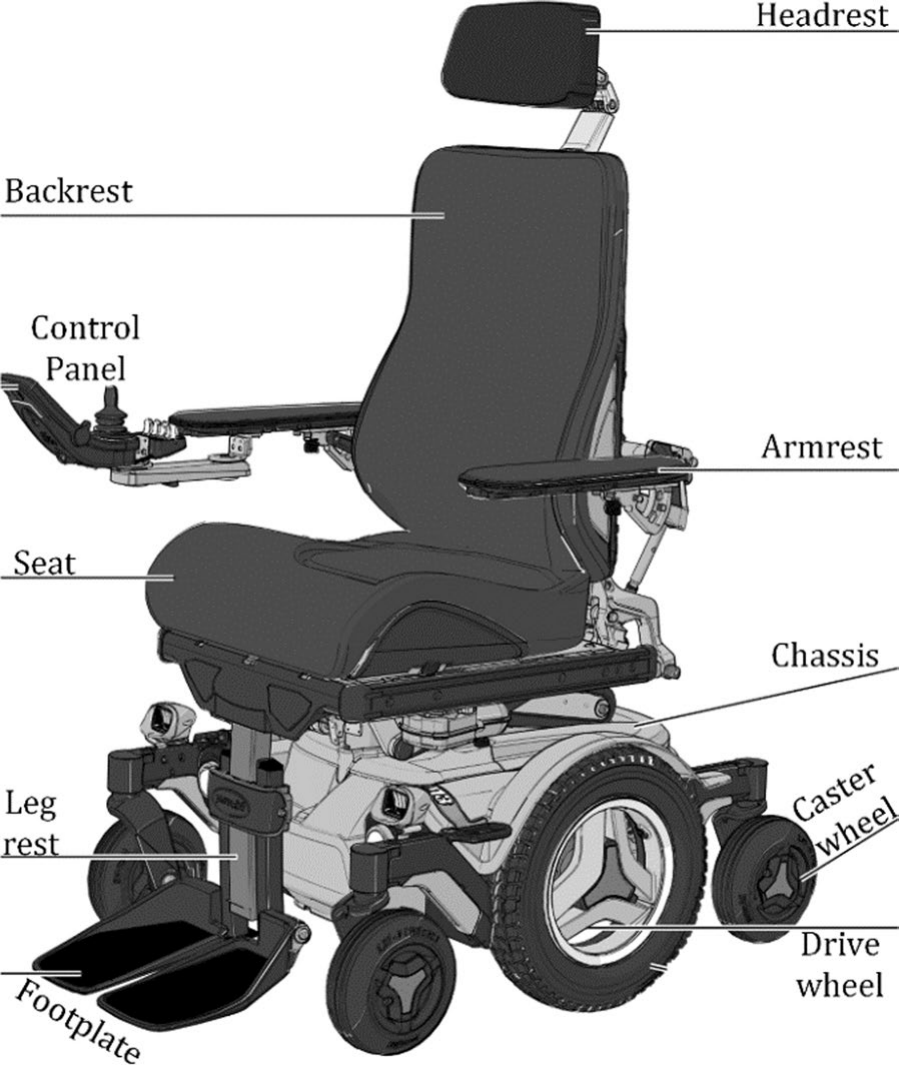
Overview of Permobil M3 corpus

Permobil M3 Corpus power wheelchair [41] comes with a carefully engineered suspension to help maintain better positioning, feel more secure, and reduce driving fatigue. M3 Corpus power wheelchair provides improved drive performance and a more consistent driving experience for all users. M3 Corpus offers 12″ of seat elevation and 20º Active Reach Technology that gives additional comfort experience and maintains full suspension at any elevation, seating position, or driving speed. Permobil M3 is incredibly reliable at low speeds, in tight areas, or using various alternative drive controls. A hardware interface module that converts the wheelchair electrical interface signals, available through a DB-9 connector in the chair electrical control module, to USB interface signals. An overview of the Permobil M3 corpus is shown in Fig. 2.

#### Eye-tracker

**Fig. 3:**
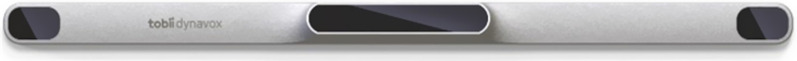
PCEye5 eye tracker

For eye-tracking control, PCEye5 from Tobii (see Fig. 3) [42] is used. The PCEye5 is a Tobii EyeChip™ with a fully embedded processing clip-on eye tracker that lets one access and controls a computer using only one's eyes. A user can select commands by dwelling, zooming, and clicking a switch by looking at the computer screen. Alternatively, a user can fully control the mouse cursor directly with eye movements. The eye tracker can be moved between different computers, and the PCEye5 is operated mainly by the user by using their eyes. It's workspace can be calibrated against each user's eye movements. The PCEye5 is attached to its Magnetic Mounting Plate and to a computer device using mountings. It is designed to work optimally when it is parallel to the user's eyes at a distance from about 50–95 cm (20 to 37 inches. Tobii Dynavox eye trackers allow easy calibration for both advanced and novice eye-tracking users. Once the PCEye is calibrated to the user’s eyes, it allows to hit even the smallest targets on the screen with almost pixel precision because of the zoom functionality of Computer Control software.

### Theoretical analysis

#### Dynamics of robotic arm

This section presents the mathematical formulation of direct kinematics, which calculates the robot’s end-effector position given the joint angles. To this end, we consider the Denavit–Hartenberg (DH) parameters for xArm-6 as presented in Table 1.

**Table 1:** Denavit–Hartenberg parameters for xArm-6

$i$	${a}_{i}$	${\alpha }_{i}$	${d}_{i}$	${\theta }_{i}$
$1$	$0$	$0$	${L}_{1}$	${\theta }_{1}$
$2$	$0$	$-\pi /2$	0	${\theta }_{2}{+}^{0}{\theta }_{2}$
3	${L}_{2}$	$0$	0	${\theta }_{3}{+}^{0}{\theta }_{3}$
4	${L}_{3}$	$-\pi$	${L}_{4}$	${\theta }_{4}$
5	0	$\pi /2$	0	${\theta }_{5}$
6	${L}_{5}$	$-\pi /2$	${L}_{6}$	${\theta }_{6}$

Here, *ɑ*_*i*_ is the lengths of the common normal, α_*i*_ is the angle about common normal, *d*_i_ is the offset along previous *z* axis, and ${\theta }_{i}$ represents the joint angles. Note that the terms ${L}_{i}$ represents the length of the $i$ link, and ${}^{0}{\theta }_{i}$ represents the offset of the $\mathrm{i}$ ${\uptheta }_{\mathrm{i}}$ angle. The values of those variables are presented in Table 2.

**Table 2:** Dimensional parameters of xArm-6

$L1$	${L}_{2}$	${L}_{3}$	${L}_{4}$	${L}_{5}$	${L}_{6}$	${}^{0}{\theta }_{2}$	${}^{0}{\theta }_{3}$
267 mm	289.49 mm	77.5 mm	342.5 mm	76 mm	97 mm	− 1.3849 rad	1.3849 rad

Additionally, the terms ${a}_{i}, {\alpha }_{i}, {d}_{i},$ and ${\theta }_{i}$ follow the DH convention as presented in [43]. This convention considers the following homogeneous transformation matrix:

1 $${}^{i-1}{T}_{i} =\left[\begin{array}{cccc}\mathrm{cos}{\theta }_{i}& -\mathrm{sin}{\theta }_{i}& 0& {a}_{i}\\ \mathrm{cos}{\alpha }_{i}\mathrm{sin}{\theta }_{i}& \mathrm{cos}{\alpha }_{i}\mathrm{cos}{\theta }_{i}& -\mathrm{sin}{\alpha }_{i}& -{d}_{i}\mathrm{sin}{\alpha }_{i}\\ \mathrm{sin}{\alpha }_{i}\mathrm{sin}{\theta }_{i}& \mathrm{sin}{\alpha }_{i}\mathrm{cos}{\theta }_{i}& \mathrm{cos}{\alpha }_{i}& {d}_{i}\mathrm{cos}{\alpha }_{i}\\ 0& 0& 0& 1\end{array}\right]$$

where ${}^{i-1}{T}_{i}$ represents the transformation between coordinate frame $i$ relative to coordinate frame $i-1$, according to Fig. 1. Ultimately, the position and orientation are obtained by applying Eq. (1). We calculated the end-effector homogeneous transformation matrix as follows:

2 $${}^{0}{T}_{6}={}^{0}{T}_{1}{}^{1}{T}_{2}{}^{2}{T}_{3}{}^{3}{T}_{4}{}^{4}{T}_{5}{}^{5}{T}_{6}$$

**Fig. 4:**
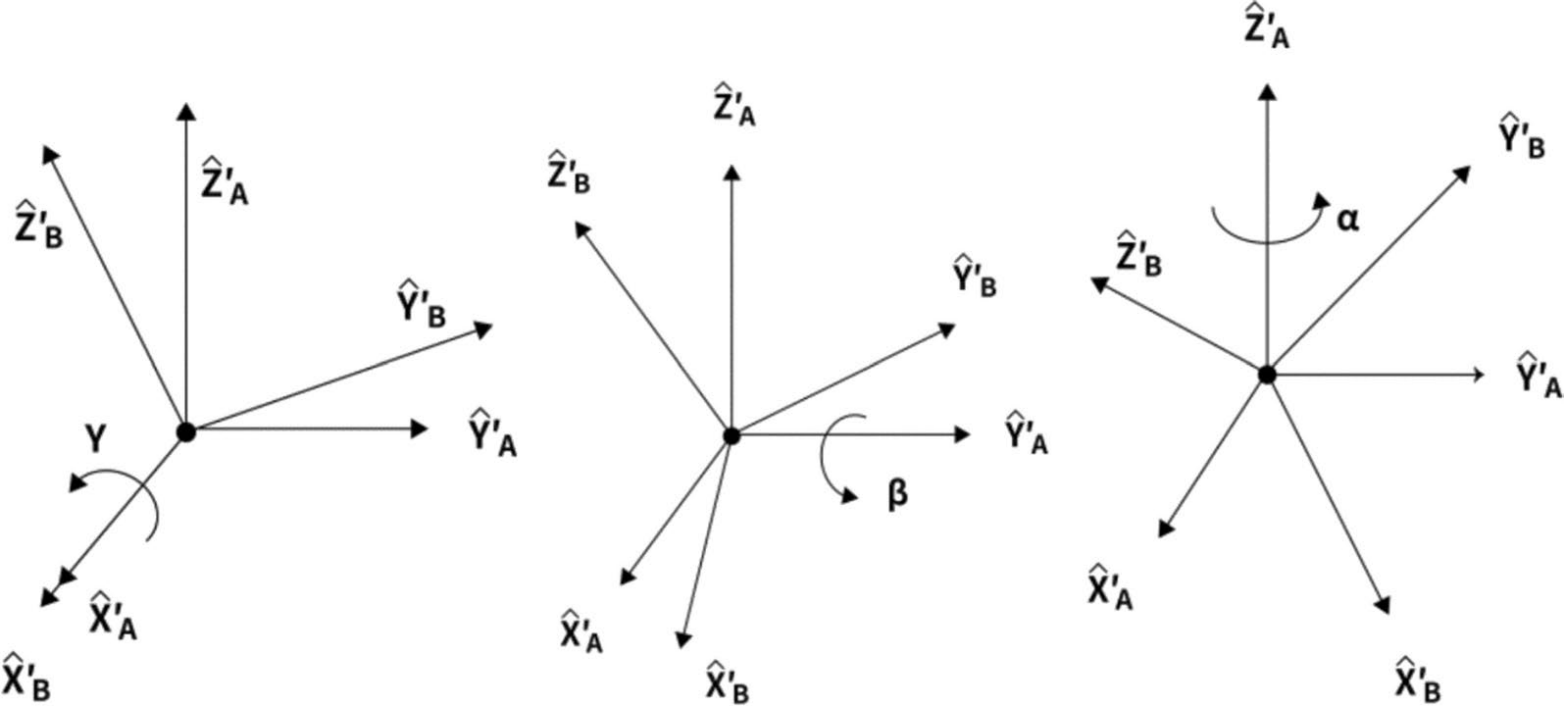
Roll, pitch, and yaw angle

Roll, Pitch, and Yaw sequentially rotate around the XYZ of the selected coordinate system. The following describes the roll, pitch, and yaw orientation representation of frame{B} relative to frame {A}: For example, the coordinate system of frame {B} and a known reference coordinate system {A} are first superposed. First rotate {B} around $\widehat{{X}_{A}}$ by γ, then around $\widehat{{Y}_{A}}$ by β, and finally around $\widehat{{Z}_{A}}$ by α. Each rotation is around a fixed axis of the reference coordinate system {A}. This method is called the XYZ fixed angle coordinate system, and sometimes they are defined as the roll angle, pitch angle, and yaw angle which is shown in Fig. 4. The equivalent rotation matrix is represented through Eq. (3).

3 $${}_{B}{}^{A}{R}_{XYZ}\left(\gamma ,\beta ,\alpha \right)={R}_{Z}(\alpha ){R}_{Y}{\left(\beta \right)R}_{X}(\gamma )$$

#### Inverse kinematics

Inverse Kinematics was performed using the gradient descent method (see Algorithm 1) [44]. For this method, the cost function is the Euclidean distance between the current end-effector position and the target end-effector position. The learning rate used in each iteration is adaptive and is a function of the cost function value. In this way, gradient descent will take bigger steps when the cost function is large and smaller steps when the cost function is small.

**Figure:**
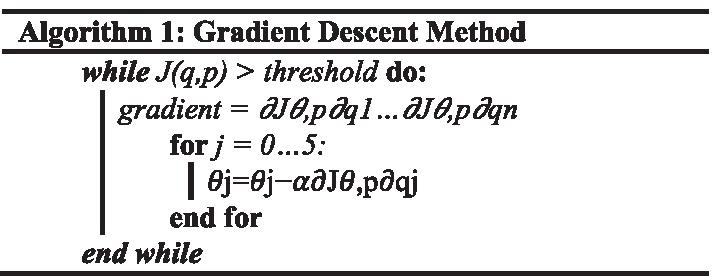

Where, *q* is the current joint angles, *p* is the target position, *J(q, p)* is the cost function defined as the distance between end-effector position and target position, and ***α*** is the learning rate.

#### Workspace consideration

**Fig. 5:**
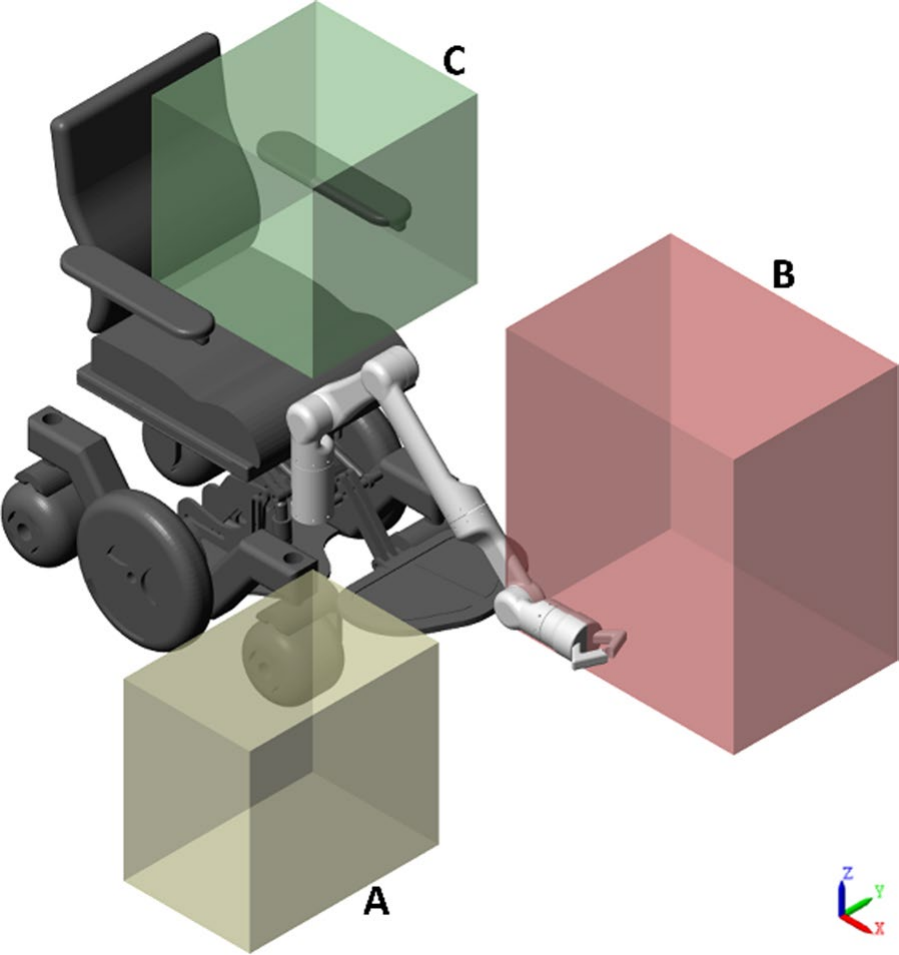
Considered workspace for daily living activities

To fulfill the activities of daily living, we considered three required workspaces. Considered workspaces are shown in Fig. 5. Each workspace has a preferred orientation of the end-effector due to its location and the activities to perform. For example, workspace C is near to the individual. Within this workspace, the robot must perform activities associated with holding or maneuvering objects. Hence, the preferred orientation is aligned with the positive y-axis. Likewise, workspace A has the preferred direction is the negative z-axis, owing to the activities that imply pick objects from the ground. Finally, workspace B is far from the wheelchair. Hence, its main direction is the positive x-axis.

#### Control architecture

**Fig. 6:**
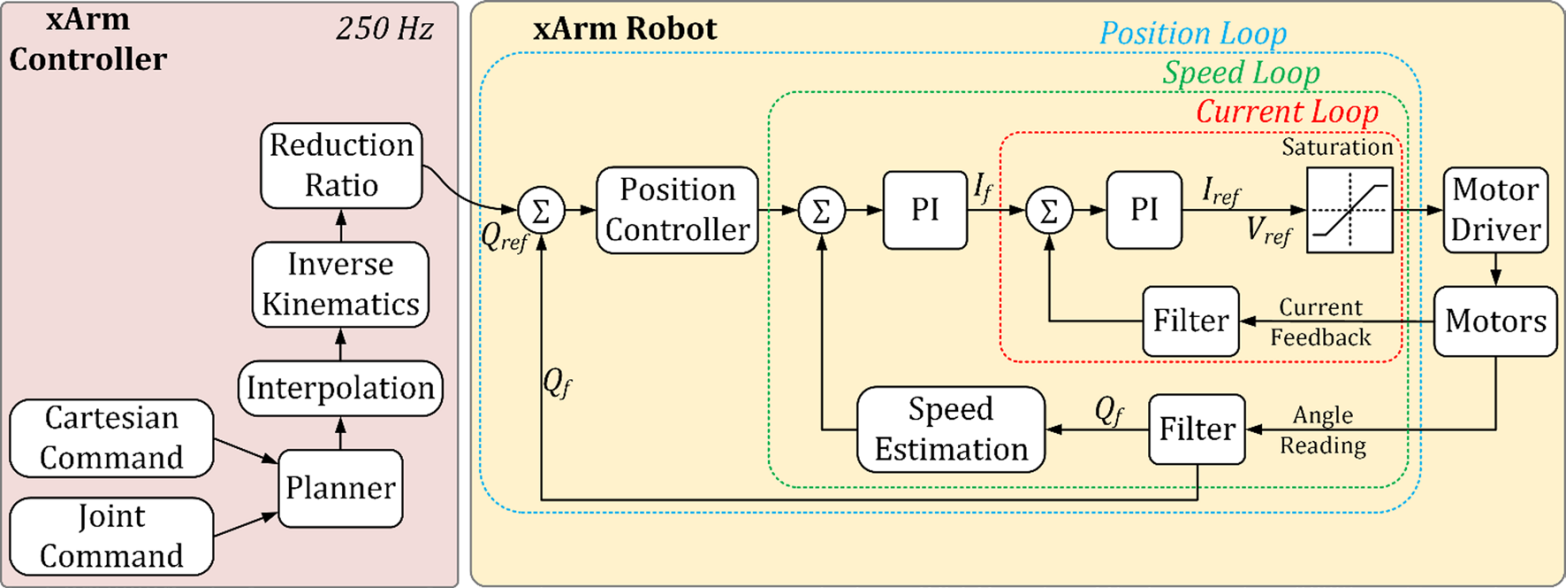
Control architecture of the system

Control architecture for the eye-gaze control system is depicted in Fig. 6. The joints' torque commands and the cartesian commands are the output of the xArm controller. However, the torque commands are converted to motor currents and finally to reference voltage as voltage value is the drive command for the motor drivers. The controller updates the torque commands every 4 ms and is executed in xArm controller. Furthermore, to realize the real-time control of the system, and also to ensure the right control torque commands were sent to the joints as well as the reference voltage commands for the drivers, we have also added a proportional-integral (PI)controller to minimize the differences between desired and measured currents The current signals measured from the current monitor output of motor drivers are sampled at 0.1 ms, and are then filtered with a 2nd order filter with a damping factor ζ = 0.90 and natural frequency ω_0_ = 3000 rad/s prior to being sent to the PI controller. This control architecture includes combination of three types of control loops: a position loop, a speed loop, and a current loop (see Fig. 6).

The primary goal of the current loop is to control torque, which influences speed, and therefore, position. The current loop is nested inside the speed loop, making current the innermost loop, with the speed loop in the middle, and the position loop being the outermost loop. The current loop here is a PI controller with both proportional and integral gains. Current control parameters are set for tuning the current control loop.

On the other hand, the speed loop compares the commanded speed to the actual speed via an encoder and issues commands to increase or decrease the motor's speed accordingly. The speed loop is also a PI controller, with a proportional gain and an integral gain to determine the correction command. The amount of proportional gain is directly proportional to the error, while the integral gain increases over time and is used to "push" the steady-state error to zero. The position loop determines the position tracking error (i.e., the deviation between the actual and desired commanded positions) and issues updated position commands to reduce or eliminate the position tracking error. In this cascaded system, the position loop used only a proportional gain.

### Graphical user interface development

User Interface is built with virtual buttons with python using PyQt5 and integrated with multithreading, allowing sending the commands from virtual buttons to the controller simultaneously. For better eye tracking, we used Tobii PCEye 5 hardware with their integrated software system. We used it with the latest Microsoft Surface Pro 7, mounted with wheelchair-using mounting brackets. We are using computer control software for tracking eye movement and operating the computer. For better performance, we have to calibrate it first. Then we use the gaze control cursor for the left mouse button click, which works both in dual time and continuous mode.

**Fig. 7:**
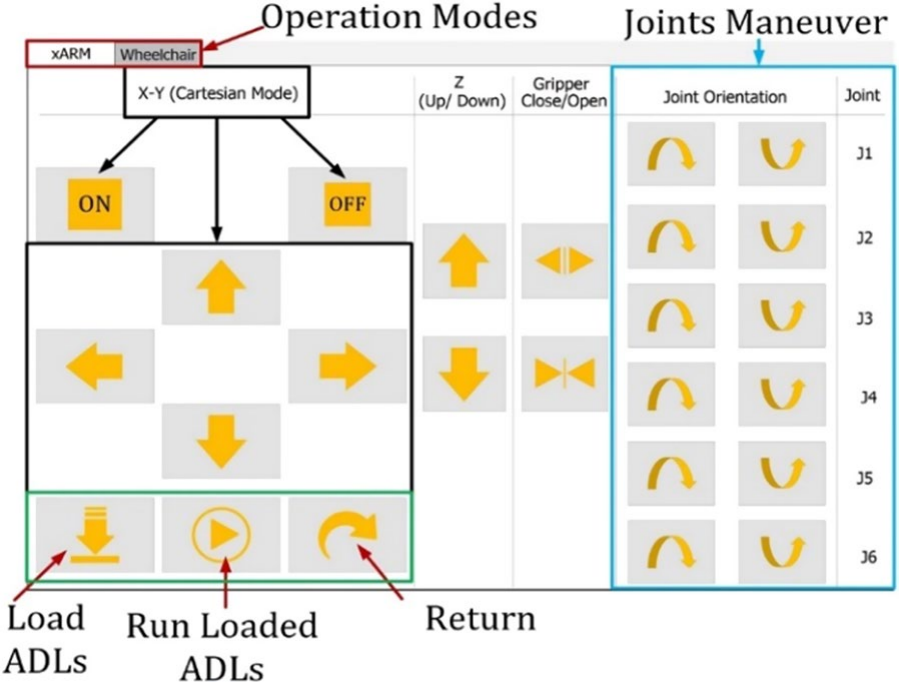
Graphical user interface for robotic arm control

At first, we started using our system with a user interface. We faced some issues that included too many buttons, small button size for triggering with eye gaze control, and the interface's complexity. After that, we updated our graphical user interface, which is much simpler and easier to understand for everyone. We have created a graphical user interface for interacting with the wheelchair and xArm. In addition, we have added a tabbed view for selecting the xArm and wheelchair mode. Figure 7 shows the updated graphical user interface to control the robotic arm. This was needed for simple button representation and ease of usage.

The xArm tab is divided into three different modes of operation. For operating the xArm we have added some buttons for the functionalities of Cartesian mode, Gripper movement, Pretrained ADLs, and Joint maneuvering. In cartesian mode, the buttons control the arm in XYZ axis and open and close the gripper. For more effective and efficient control, we added buttons for maneuvering the joints individually. For repetitive ADL tasks, we added buttons to load and run predefined trajectories. Using the return button, the end-effector can move between two specific target positions following the same trajectory.

**Fig. 8:**
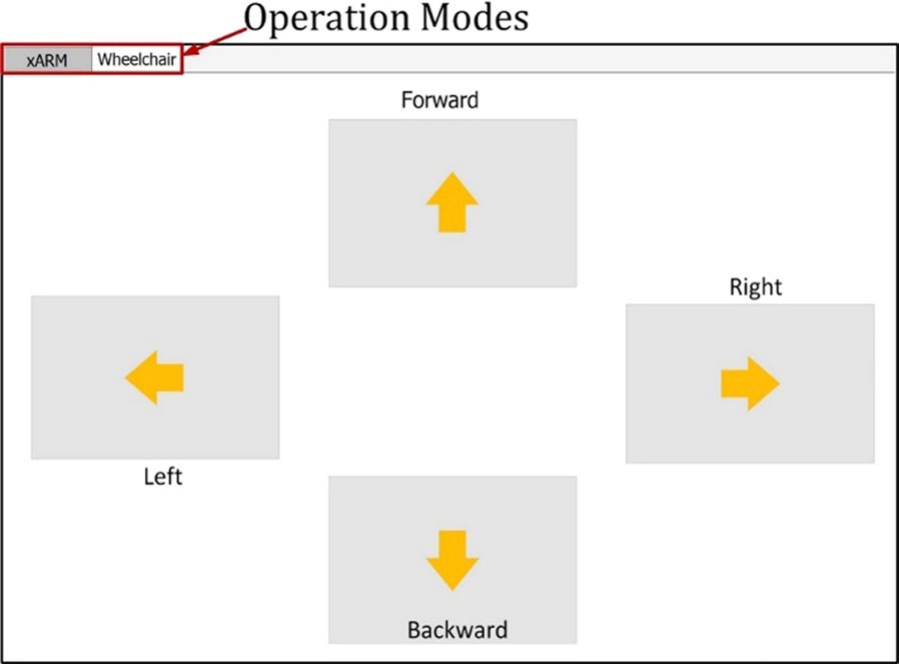
Graphical user interface for controlling the wheelchair

Again, for moving the wheelchair, we have added virtual buttons. In the wheelchair tab of the interface, four buttons are placed, which is triggered through the left mouse click, and this click is done through eye gaze dwell time. If the mouse cursor is on the button after the dwell time wheelchair will go in that specific direction; and the wheelchair will stop if the cursor moves out from the button. Figure 8 shows the user interface for controlling the wheelchair using eye gaze control.

### Setting of the study

**Fig. 9:**
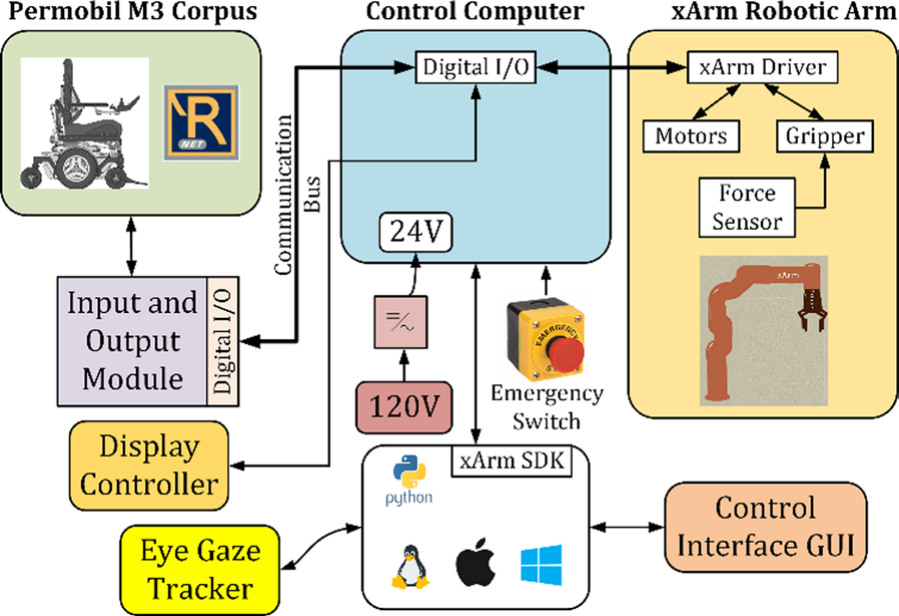
Block diagram of the experimental setup

Figure 9 shows the components and connections of the robotic-assisted power wheelchair with the control architecture. The green section is composed of the Permobil M3 corpus power wheelchair with its electronics that use the R-net control system that manages the inputs and output to control and share the variables of the wheelchair. Using R-net[45], the Input–Output Module (IOM), purple box, takes the joystick values. Through a D-Sub 9 Pin, it sends logical values (0 or 1) for each direction sent by the input device in the chair, or in our case, it receives logical values from an external computer to move the wheelchair. The robotic assistive arm consists of its drivers, motors, actuators, and sensors, and this is a self-contained device over a designed data. Power cable gets the control signals and shares status data (position, speed, acceleration, torque, current consumption, etc.) to an external computer.

The white box shows the user application layer. This can be run in any common operatives system (OS) like Linux, macOS, and Windows. The programs in this layer are based on Python programming language and a Software Development Kit (SDK) that came with the robotic assistive arm. From this layer, the control signals to move the arm and the power wheelchair are sent. It also can read the variables of the whole system to do an appropriate control. The program in this layer was designed to be used with an Eye Gaze Tracker to allow patients with restricted mobility to access all the old and new system functionalities.

**Fig. 10:**
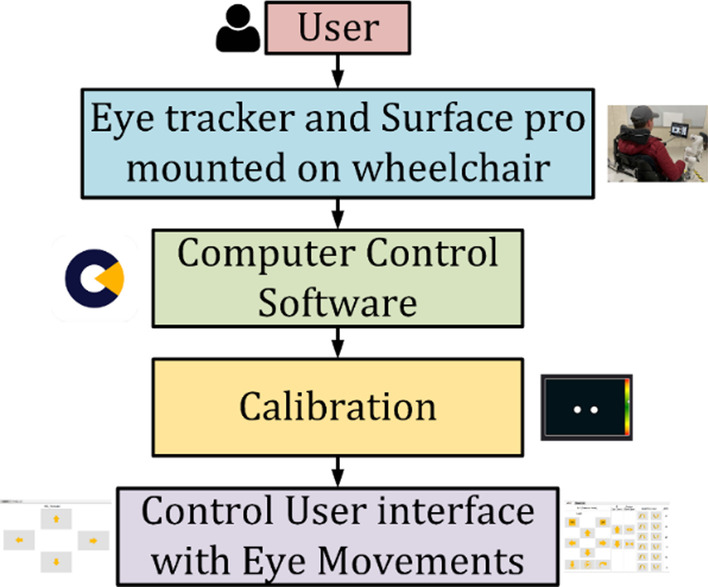
Flowchart of the experiment

Integrated system with eye tracker, developed circuitry, and a software system that seamlessly allows users to control wheelchair and assistive robotic arms. The blue box represents the control computer. It handles the communications of the whole system, reading and sending data to the power wheelchair with its General-Purpose Input Output (GPIO), sending and receiving data from the application layer over ethernet communication, and manipulating the robotic arm over a closed-loop control. This box gets the main power supply from the Alternative Current (AC) connection and regulates it to a 24 V supply and logic for its references. It also has an emergency stop button in case of an undesired situation. The flowchart in Fig. 10 shows that the user needs to calibrate the eye tracker in computer control software.

## Results

The study conducted in this manuscript was approved by UWM IRB (21.310.UWM). After getting the IRB approved, for validating the developed system, we recruited healthy subjects to do the activities of daily living. Socio-demographic profiles of the participants are presented in Table 3.

**Table 3:** Description of the profiles of participants (N = 10)

Characteristics	Value
*Age (years)* (Mean ± Standard Deviation) (n = 10)	27.8 ± 2.95
*Gender*	
Male	9
Female	1
*Civil status*	
Single	7
Married	3
*Health status*	
Healthy	10
Person with disability	0

**Fig. 11:**
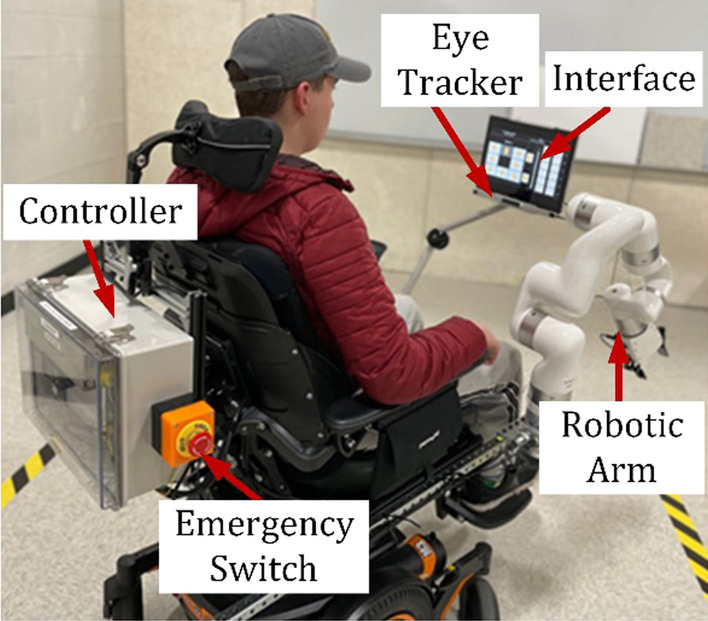
A user is sitting in a wheelchair and using the system

The xArm is mounted with the side rail of the Permobil power wheelchair. Figure 11 shows the experimental setup with the robot mounted on the wheelchair. We made accounts for each participant in our system and calibrated the eye tracker using the computer control software to use the developed graphical user interface to control the wheelchair and robotic arm performing activities of daily living. As a safety measure, we constrained the robot workspace to avoid contact between the robot and the participants.

**Fig. 12:**
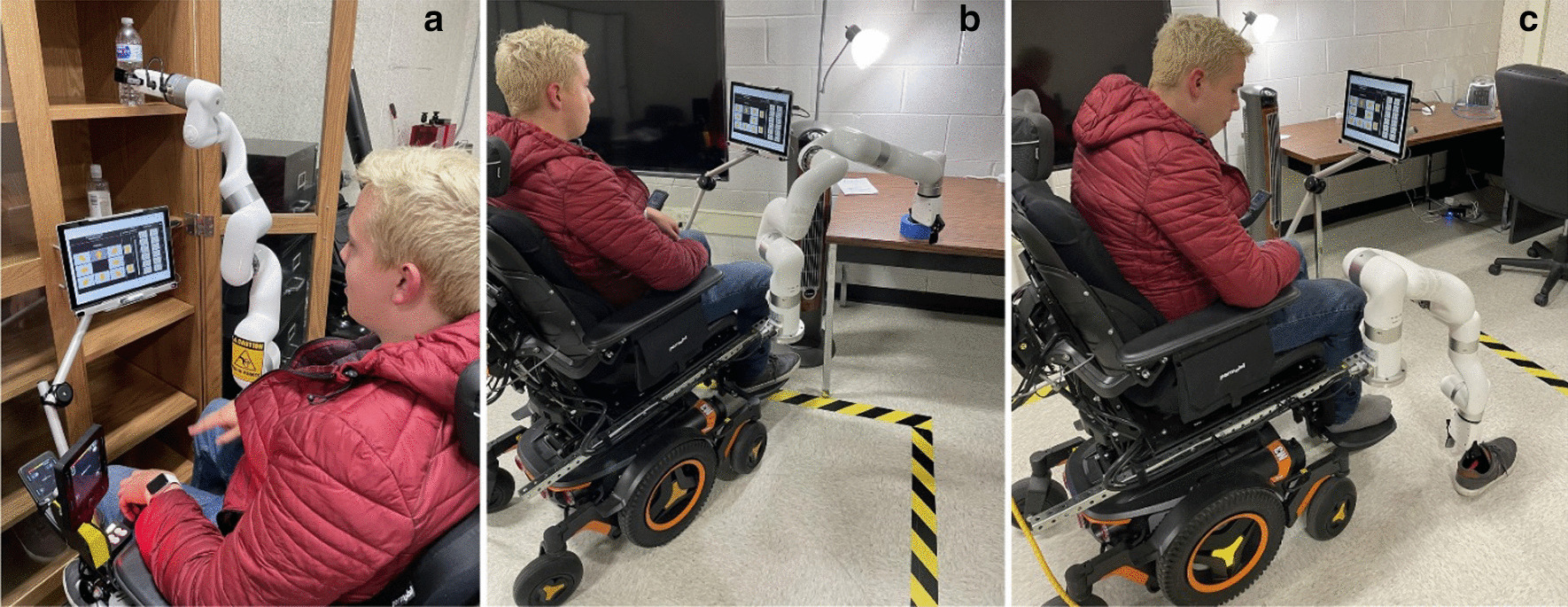
Activities of daily living experiment with a healthy subject. From the left, **a** getting something from the upper shelf, **b **picking objects from the table, and **c** picking things from the ground

**Fig. 13:**
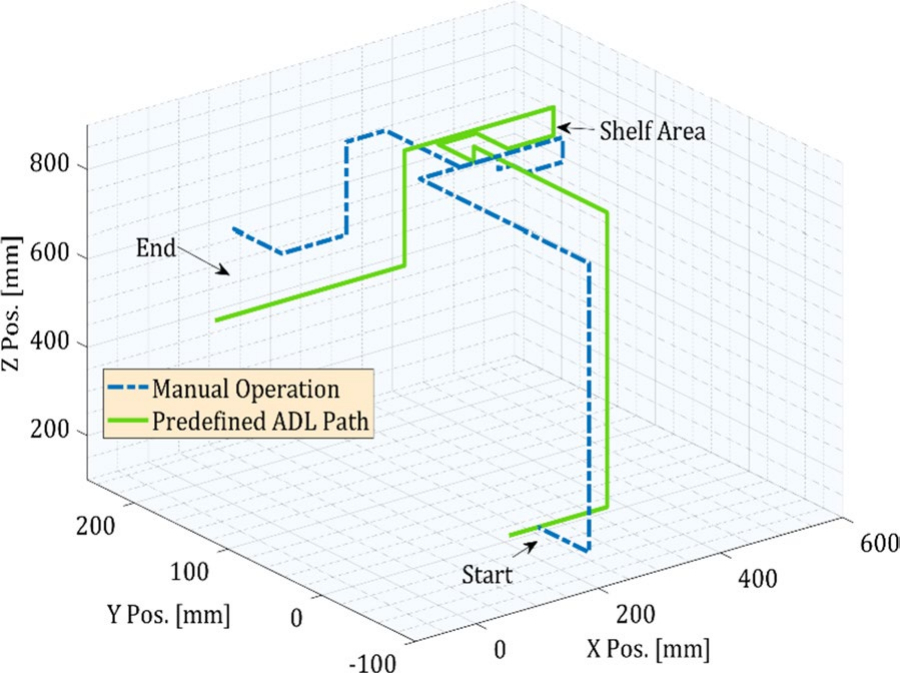
Trajectories of picking an object from a shelf using cartesian mode as well as following a predefined path

**Fig. 14:**
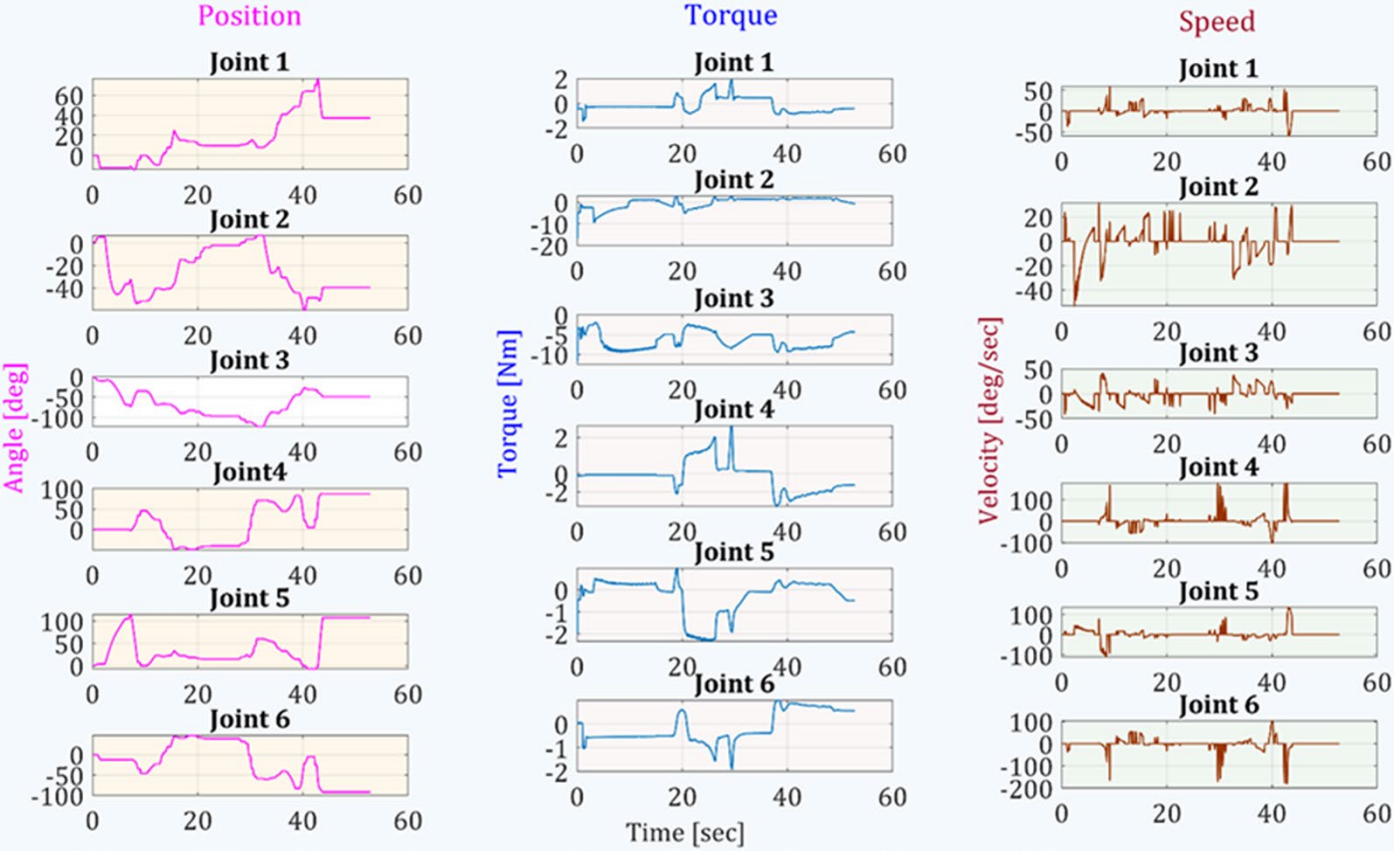
Joint angles, torques, and speed observation while picking an object from a shelf

As activities of daily living, we selected picking objects from the upper shelf, picking an object from a table, picking an object from the ground. We recorded the data for both manual operations of the robotic arm and pre-recorded trajectories of these activities of daily living. Cartesian mode is used for the manual operation of the robotic arm. Still, for predefined ADL trajectories, the pickle file is loaded and executed through the graphical user interface to complete the task. Figure 12 shows the example of these ADL tasks performed using eye gaze control. The object on the shelf was five feet from the ground, and the object on the table was two and a half feet from the ground. Figure 13 shows the end-effector trajectories while performing an ADL picking object from an upper shelf in manual cartesian mode operation and previously saved path. From both trajectories, it can be concluded that manual cartesian manipulation is traveling some extra distance than the predefined ADL path. The joint angles, torques, and speed of each joint are represented in Fig. 14. Each joint begins to move at the initial angle for this specific task and stops at the final angle. From the initial position of the end effector to the final position, most of the load was imposed on joint two and joint three. We can also observe the speed fluctuations over the course due to the switching frequencies of the control.

**Fig. 15:**
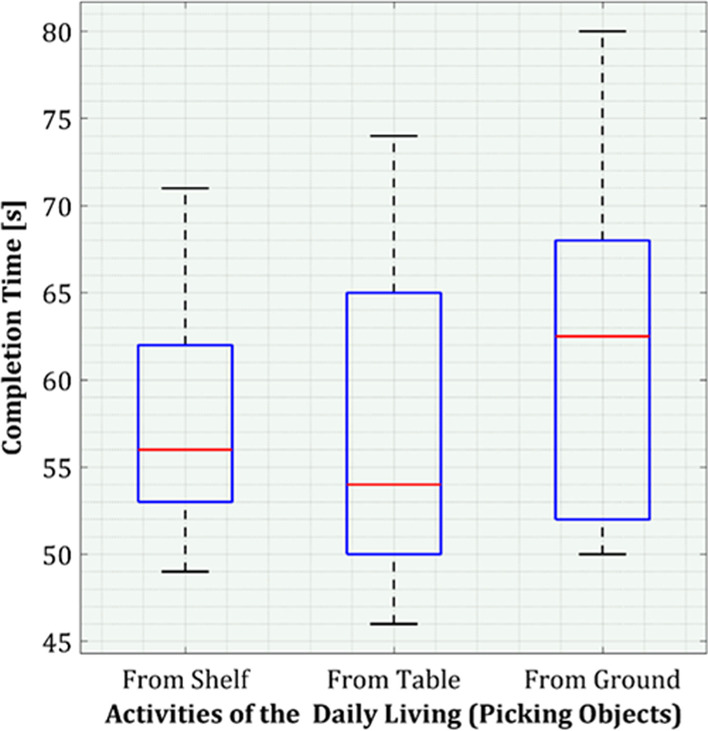
Completion time analysis of activities of daily living

This study yields ten samples from 10 participants from 3 types of tasks. Figure 15 shows the distribution of completion time as a box plot. For picking an object from a shelf, the minimum time required is 53 s, the maximum time required is 71 s, and the median for this task is 56 s. Also, for picking an object from a table, the minimum time required is 46 s, the maximum time required is 74 s, and the median for this task is 54 s. Similarly, for picking an object from the ground, the minimum time required is 50 s, the maximum time required is 80 s, and the median for this task is 63 s. Before performing all these tasks, participants practiced for around 15 min. After completing all the tasks, we got positive responses from the participants, and they performed all tasks with ease. Table 4 shows the overall experience of 10 healthy participants. Table 5 presents the performance of the proposed method in comparison with other studies in terms of calibration time and accuracy. The accuracy of the proposed method is about ${0.8}^{o}$. Furthermore, the calibration time for the proposed method is minimal among all the other methods.

**Table 4:** Overall experience using assistive robot

Item	Question	Avg. Score (0–5) (N = 10)
1	How do you rate this Assistive Robot (overall satisfaction)	4.65
2	How do you rate the comfort of using this Assistive Robot?	4.72
3	How do you rate the ease of maneuverability of this Assistive Robot?	4.58
4	How did this Assistive Robot assist you with Activities of Daily Living (ADL)?	4.88

**Table 5:** Performance of different methods compared to the proposed one

Methods	Calibration time (minutes)	Accuracy (degrees)	Approach
[46]	3	1.28	Web camera, free head movement
[47]	1	0.6	Headset with optical tracker
[48]	3	2	Depth camera, free head movement
[49]	0.6 (36 s)	0.24	Eyeglasses with an optical tracker, free head movement
Proposed method	0.5 (30 s)	0.8	Tobii PCEye5 eye tracker, free head movements

## Discussion

This proposed method is for individuals with higher upper extremity motor dysfunctions to do activities of daily living with a wheelchair-mounted robotic arm. Because of the higher degree of the upper extremity, they cannot control the wheelchair or the robotic arm with other standard input devices such as finger or chin-controlled joystick. The developed control system and the interface are validated through the experiments with the healthy participants. Our experiments involved different essential activities of daily living and controlling the wheelchair with the same interface, which is also necessary for the mobility independence of people with disabilities. The overall experience results obtained from the participants indicate a promising solution for the individuals who are struggling with or unable to do their primary day-to-day tasks for their motor dysfunctions.

Participants provided positive feedback about the eye-gaze interface for its user-friendly design. For the large virtual buttons, they could control it with ease. The tabbed view feature in the interface allowed us to add bigger buttons for controlling both the wheelchair and the robotic arm. We acknowledge that it is difficult to make generalized statements about the findings with only the healthy participants. In future studies, people with upper limb mobility impairments are necessary to derive robust conclusions about the proposed method. Also, there are some uncertainties with the participants’ feedback as it is not objective.

Additionally, as our method involves eye movement, the proposed method can only be used for a user who has intact eye movement. To make this control system more efficient and user-friendly in the next phase, we will add object detection features. Object detection and recognition features of computer vision will aid the user in manipulating objects involved with activities of daily living more easily.

## Conclusion

The objective of this work was to develop a control architecture to assist people with disabilities using an eye gaze interface to control a wheelchair and wheelchair-mounted robot arms for their daily living activities. To accomplish this task, we first develop a graphical user interface based on wheelchair and robotic arm control commands to control manually in cartesian mode and follow the predefined trajectory of specific ADLs. Then, we solved the inverse kinematic of the robotic arm using the gradient descent algorithm. Finally, we evaluated the control system involving the eye gaze tracker in a human–robot collaboration with ten healthy participants. We constrained the robot workspace for safety measures. This project conforms to how eye gaze helps to manipulate assistive robotics. Our future direction for this project will be improving the robotic control architecture to reduce task completion time. Moreover, we will evaluate our developed system further with people with upper mobility impairments.

## Data Availability

The datasets generated during and/or analyzed during the current study are not publicly available due to the conditions of the funding source but are available from the corresponding author on reasonable request.
